# Quantifying risk factors and potential geographic extent of African swine fever across the world

**DOI:** 10.1371/journal.pone.0267128

**Published:** 2022-04-21

**Authors:** Dong Jiang, Tian Ma, Mengmeng Hao, Fangyu Ding, Kai Sun, Qian Wang, Tingting Kang, Di Wang, Shen Zhao, Meng Li, Xiaolan Xie, Peiwei Fan, Ze Meng, Shize Zhang, Yushu Qian, John Edwards, Shuai Chen, Yin Li

**Affiliations:** 1 Institute of Geographic Sciences and Natural Resources Research, Chinese Academy of Sciences, Beijing, China; 2 College of Resources and Environment, University of Chinese Academy of Sciences, Beijing, China; 3 Centre for Tropical Medicine and Global Health, Nuffield Department of Medicine, University of Oxford, Oxford, United Kingdom; 4 School of Urban Planning and Design, Peking University Shenzhen Graduate School, Shenzhen, Guangdong, China; 5 School of Geographic Sciences, Nantong University, Nantong, China; 6 Computer Network Information Center, Chinese Academy of Sciences, Beijing, China; 7 School of Veterinary Medicine, Centre for Biosecurity and One Health, Murdoch University, Perth, Australia; 8 Commonwealth Scientific and Industrial Research Organisation, Brisbane, Australia; University of Lincoln, UNITED KINGDOM

## Abstract

African swine fever (ASF) has spread to many countries in Africa, Europe and Asia in the past decades. However, the potential geographic extent of ASF infection is unknown. Here we combined a modeling framework with the assembled contemporary records of ASF cases and multiple covariates to predict the risk distribution of ASF at a global scale. Local spatial variations in ASF risk derived from domestic pigs is influenced strongly by livestock factors, while the risk of having ASF in wild boars is mainly associated with natural habitat covariates. The risk maps show that ASF is to be ubiquitous in many areas, with a higher risk in areas in the northern hemisphere. Nearly half of the world’s domestic pigs (1.388 billion) are in the high-risk zones. Our results provide a better understanding of the potential distribution beyond the current geographical scope of the disease.

## Introduction

African swine fever (ASF) is an acute, contagious swine disease that is becoming a global threat due to its devastation on pig production [1]. Its causative pathogen is African swine fever virus, which is a DNA virus belonging to the family *Asfarviridae*, genus *Asfivirus*. The virus can survive for a long time in the contaminated environment [2, 3], which leads to a broad range of clinical signs in sick domestic pigs, such as sudden death, high fever, haemorrhage, anorexia, dyspnea and vomiting, with the lethality rates approaching 100% when highly pathogenic strains infect pigs [4]. ASF is listed by the World Organization for Animal Health (OIE) as a notifiable animal disease (https://www.oie.int/en/disease/african-swine-fever/).

African swine fever was first reported in eastern Africa in 1921 [5]. Since then, additional cases have been reported in most of the Sub-Saharan countries [6, 7]. In 1957, ASF was found in Europe, arriving in Portugal from Angola via contaminated swill from aircraft [8]. It then continued to spread to Western European countries (i.e., Spain), the Caribbean, and Brazil. The disease was eradicated in these places in the mid-1990s through culling and movement bans of live pigs and pig products, except for the Italian island of Sardinia [9–11]. In 2007, the disease was reported in the Republic of Georgia, a transportation hub of Europe, and then it spread widely in the vast areas of Eastern Europe, including Russia (2007), Ukraine (2012), Belarus (2013), Lithuania (2014), Estonia (2014), Poland (2014), Latvia (2014), and Romania (2017), and Czech Republic (2017) [6, 12–15]. In 2018, ASF was detected in China for the first time [16]. Since then it spread to more Asian countries, including Vietnam, Cambodia, North Korea, Laos, Indonesia, Myanmar, Philippines and South Korea [17–19]. No commercial vaccines and effective treatments are available to control the disease [20, 21].

In order to predict and control for the further spread of the disease beyond the existing geographic range, predictive models linking the locations of the reported cases to environmental risk factors were adopted to improve the risk-based surveillance and control [9, 22–24]. For example, Cappai et al. combined biological and socio-economic factors with the negative binomial regression model to estimate the ASF risk in Sardinia [9]. A study conducted by Liang et al. showed that ASF is associated with precipitation based on several machine learning methods [23]. Based on the data of 98 ASF cases reported from 2018 to 2019 in China, Ma et al. employed a maximum entropy model using pig density and various meteorological covariates to identify the high risk areas for the disease outbreaks in China [24]. Although several environmental risk factors have been identified in previous studies, the difference in the risk of ASF in domestic pigs and wild boars was not assessed.

To address these limitations, we assembled contemporary records of ASF in both domestic pigs and wild boars, and paired them with a set of livestock density, anthropogenic and habitat correlates to quantify the risk factors. The risk of ASF was predicted at a global scale using a modeling framework. Additionally, we estimated the potential burden of ASF in countries, providing novel insights to inform global, regional and national health authorities who are developing control strategies for the disease.

## Materials & methods

### Data

#### ASF cases

The data on ASF cases were downloaded from the website of the EMPRES Global Animal Disease Information System (EMPRES-i) of the Food and Agriculture Organization (FAO) of the United Nations (http://empres-i.fao.org/eipws3g/), which has been designed to support veterinary services by facilitating the organization and access to regional and global disease information. The cases of ASF were collected from European Commission, FAO officers, national authorities and OIE. In this study, the ASF cases (16,550) spanned from 2005 to 2019 were used, of which 12,089 occurred in wild boars, 4,502 occurred in domestic pigs and 41 co-occurred in both wild boars and domestic swine over the world.

#### Spatial predictor variables

*Livestock factors*. Domestic pigs play a key role in the transmission of ASF [25]. Direct contact between sick and susceptible domestic pigs have been considered to be an effective and important transmission route for this disease, which are likely to be significant in the disease persistence in endemic areas as well as sporadic outbreaks or introduction into disease-free zones [26–28]. In addition, previous literature have suggested that pig density distribution is associated with the occurrence of ASF [24, 29]. Thus, in this study, the density of domestic pigs was considered to be an important livestock factor, and was downloaded from the Food and Agriculture Organization (http://www.fao.org/livestock-systems/global-distributions/pigs/en/).

*Anthropogenic factors*. Human activity (e.g. trade and travel) can lead to transmission of ASF over both short and long distances [25, 30]. African swine fever virus in infected animals and contaminated fomites or products may lead to transboundary or even transcontinental spread of ASF [31]. For example, an ASF outbreak in Georgia in 2007 was caused by improper disposal of contaminated pork meat from a ship at docks [31]. Moreover, a study conducted by Gulenkin et.al also suggested that the density of road networks was one of the risk factors for disease spread [32]. Population density and night-time lights could reflect the level of urbanization, and urban accessibility could imply the frequency of trade to some extent. In the present study, we assumed that population density, night-time lights and urban accessibility as three anthropogenic factors that may affect the disease transmission in our boosted regression tree (BRT) model. The data sets of urban accessibility, population density and night-time lights, can be obtained free from the European Commission Joint Research Centre (ECJRC) (http://forobs.jrc.ec.europa.eu/), the Socio-economic Data and Applications Center, NASA (https://earthdata.nasa.gov/eosdis/daacs/sedac), and the Earth Observation Group, NOAA (http://www.earthobservations.org) respectively.

*Habitat factors*. Habitat factors play important roles in limiting the distribution of hosts, thereby affecting the risk of disease transmission. Domestic pigs, wild boars and soft tick vectors as hosts for ASF virus, have been shown to be significantly associated with the presence ASF [25, 28, 32]. On this basis, the habitat distribution of these hosts is supposed to be influenced by climate conditions such as precipitation and temperature according to some previous literatures [24, 33–36]. In addition, land cover, elevation and NDVI also probably influence the distribution of hosts by affecting the food and habitats of hosts, for example, the bushes created habitats for ticks [37]. Hence, land cover, elevation, NDVI and climate conditions (mean temperature, water vapor pressure and annual cumulative precipitation) were supposed to be potential habitat variables for ASF presence in this study. The dataset of land cover, elevation and normalized difference vegetation index (NDVI) were obtained from NASA’s Earth Observatory Group (https://earthobservatory.nasa.gov/), the CGIAR Consortium for Spatial Information (http://srtm.csi.cgiar.org), and the Global Inventory Modelling and Mapping Studies (GIMMS) group (https://ecocast.arc.nasa.gov/). Climate data including mean temperature, water vapor pressure and annual cumulative precipitation can be downloaded from WorldClim database, version 2 (http://www.worldclim.com/).

### Modelling analysis

A ensemble BRT modeling framework, that has been successfully used for predicting potential geographic extent of several diseases (i.e., visceral leishmaniasis and scrub typhus), was adopted due to the ability to effectively capture complex response functions [38–41]. The “gbm” and “dismo” extent packages were used to perform modelling procedures based on the R v 3.3.3 programming environment. The BRT model adopted in this study can be described as follows (Eq 1):

$$f_{t}(X)=f_{t-1}(X)+\lambda \cdot \rho_{t}h(X;a_{t})\;\lambda \in (0,1]$$ (1)

L(y,f(X))=log(1+exp(−2yf(X))) (2)

Where y is the response, *X* = {*x*_1_,*x*_2_,⋯,*x_n_*} denotes livestock, anthropogenic and habitat factors, *f_t_*(*X*) refers to the mapping function from X to y during the t-th iteration, *λ* is the learning rate, *ρ_t_* is the weight parameter, *h*(*X*;*a_t_*) represents an individual tree. The parameters were estimated by minimizing a binomial loss function (Eq 2).

The assembled contemporary records of ASF cases of both domestic pigs and wild boars were rasterized to grid cells to match the spatial resolution of predictor variables (S1 Table) of approximately 5 km × 5 km. According to the contingency plan (http://www.moa.gov.cn/govpublic/xmsyj/202104/t20210429_6367009.htm) published by Ministry of Agriculture and Rural Affairs of the People’s Republic of China, regions extending 13km radially away from where ASF cases are reported are designated high-risk zones for domestic pigs, while in wild boar infection areas, they require 40km further extension. In total, we obtained 1,914 and 3,520 samples of high ASF risk for domestic pigs and wild boars respectively. In the present study, areas outside the high-risk zones are determined to be low-risk, which were used as basis for pseudo-absence samples. In order to reduce the impact of the number of pseudo-absence samples on modeling procedure, the ratio of pseudo-absences to occurrence high risk samples is set to 0.5, 0.75, 1, 1.25, 1.5 and 2, respectively [42]. For each ratio, pseudo-absences samples were selected at random, and the process was conducted 25 times. During each modeling procedure, pseudo-absences samples and occurrence high risk samples were merged into a total sample, then divided into training samples (75%) and validation samples (25%). According to the suggestion of Jiang et al. [43], the main parameters were set as follows: tree.complexity as 4, learning.rate as 0.005 and max.trees as 10,000. In order to avoid over-fitting, the 10-fold cross-validation method was employed to select the optimal number of trees for each BRT model. An ensemble BRT was constructed using 150 sub-models, which was used to generate the mean infection risk level map (risk level ranging from 0 to 1) for domestic pigs and wild boars respectively. In the present study, the area under the curve of the receiver operating characteristic plot (AUC-ROC) and the relative contribution (RC) were adopted to quantify the performance of the ensemble BRT models and the importance of spatial predictor variables, respectively.

## Results

### Global distribution of African swine fever

**Fig 1:**
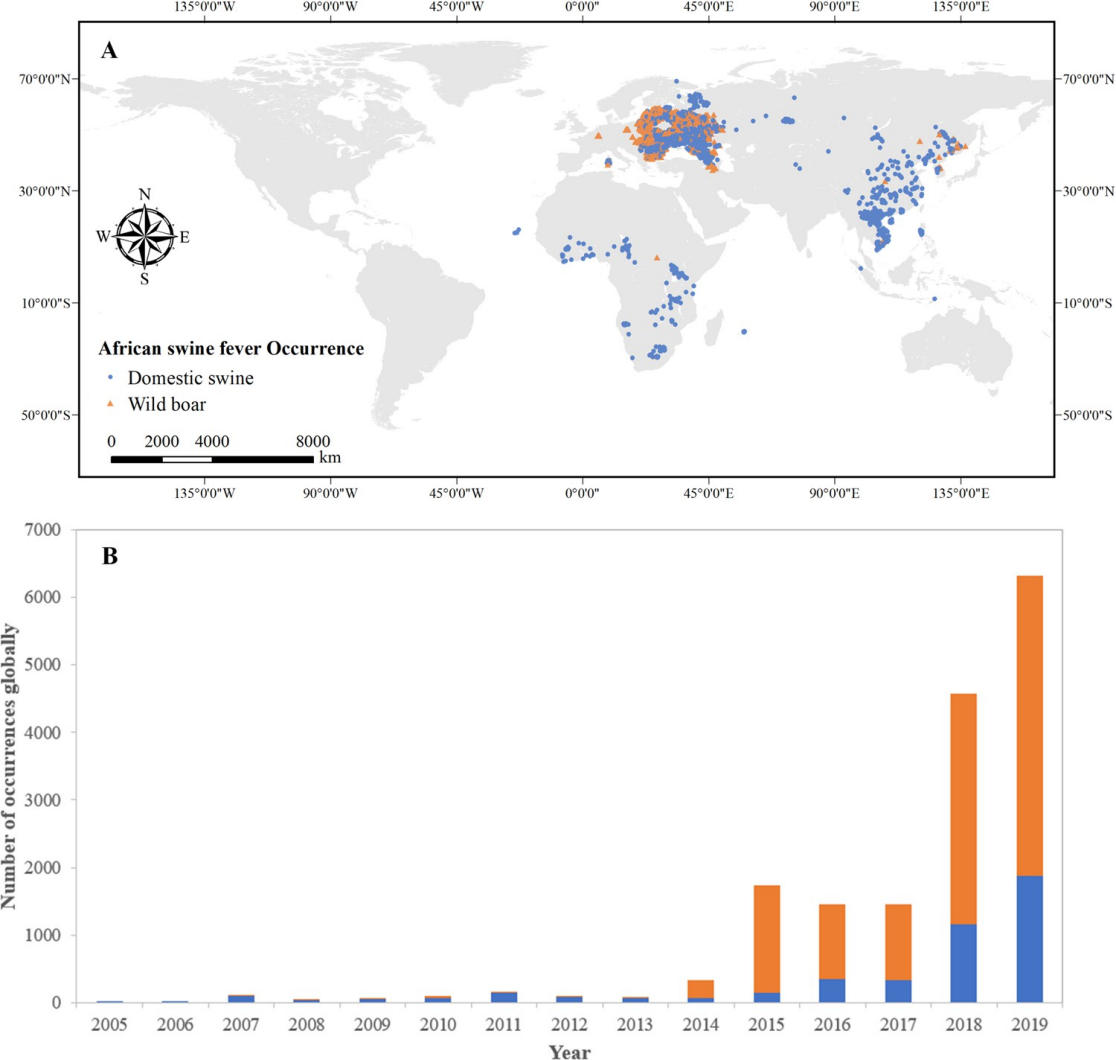
(A) The global distribution of 16,550 African swine fever cases from 2005 to 2019. Locations are classified by the type of pigs: domestic pigs (blue dots); and wild boars (yellow triangles). (B) The number of African swine fever cases globally over time (2005–2019).

Fig 1A shows the locations of all the 16,550 ASF cases spanning from 2005 to 2019, from which we could conclude that the disease mainly occurred in western Europe, Africa, and eastern Asia, affecting 52 countries. There have been cases of domestic pigs infected with ASF in 48 countries (accounting for 92% of the total number of infected countries), and wild boars infected with ASF in 23 countries (accounting for 44% of the total number of infected countries). Fig 1B shows the number of reported ASF cases globally by year (from 2005 to 2019). During this period, the number of ASF cases showed an overall increasing trend, from 3 cases in 2005 to 6,357 in 2019. Among all ASF cases, 27% of them were derived from domestic pigs, and 73% were from wild boars.

### Relative contribution of risk factors

The relative contribution (RC) of livestock, anthropogenic and habitat predictor variables were quantified by the ensemble BRT models, as shown in Table 1. For the risk of ASF derived from domestic pigs, the most noteworthy predictor variables were, in decreasing order of RC values, domestic pig density (RC 43.807%), water vapor pressure (RC 13.678%), urban accessibility (RC 11.512%), land cover (RC 10.255%), mean temperature (RC 6.173%) and elevation (RC 4.483%). Whilst annual cumulative precipitation (RC 2.855%), population density (RC 2.811%), NDVI (RC 2.371%) and nighttime light (RC 2.054%) did not substantially contribute to the ensemble BRT models fitted from ASF cases in domestic pigs. For the risk of spread of ASF derived from wild boars, water vapor pressure (RC 56.388%), mean temperature (RC 28.547%), NDVI (RC 4.138%), urban accessibility (RC 3.803%) and elevation (RC 3.097%) were the main predictor variables, and the RC rate of the remaining variables was less than 3%. In total, livestock factor (RC 43.807%) had high relative influence statistics for the spread risk level of ASF derived from domestic pigs, while the RC of habitat predictor variables (95.015%) was higher in the ensemble BRT models fitted from wild boars ASF cases. In the present study, correlation matrix and variance inflation factor (VIF) were calculated for collinearity test, as shown in S2 and S3 Tables. Generally, the values of VIF are less than 10, indicating that there is no collinearity between independent variables.

**Table 1:** Relative contribution of livestock, anthropogenic and habitat covariates derived from the ensemble BRT models.

	Mean relative contribution ± SD	
	Domestic pigs	Wild boars
**Livestock†**	**43.807**	**0.822**
Domestic pig density	43.807 ± 6.533	0.822 ± 0.248
**Anthropogenic†**	**16.377**	**4.163**
Urban accessibility	11.512 ± 2.904	3.803 ± 2.391
Population density	2.811 ± 2.735	0.275 ± 0.096
Nighttime light	2.054 ± 0.725	0.085 ± 0.045
**Habitat†**	**39.816**	**95.015**
Water vapor pressure	13.678 ± 3.921	56.388 ± 6.339
Land cover	10.255 ± 4.126	2.191 ± 2.318
Mean temperature	6.173 ± 1.827	28.547 ± 2.848
Elevation	4.483 ± 1.496	3.097 ± 0.896
Annual cumulative precipitation	2.855 ± 0.757	0.655 ± 0.286
NDVI	2.371 ± 0.573	4.138 ± 1.099

* BRT: boosted regression tree; NDVI: normalized difference vegetation index.

†Sum of the relative contribution for each category.

### Global spread risk of ASF

**Fig 2:**
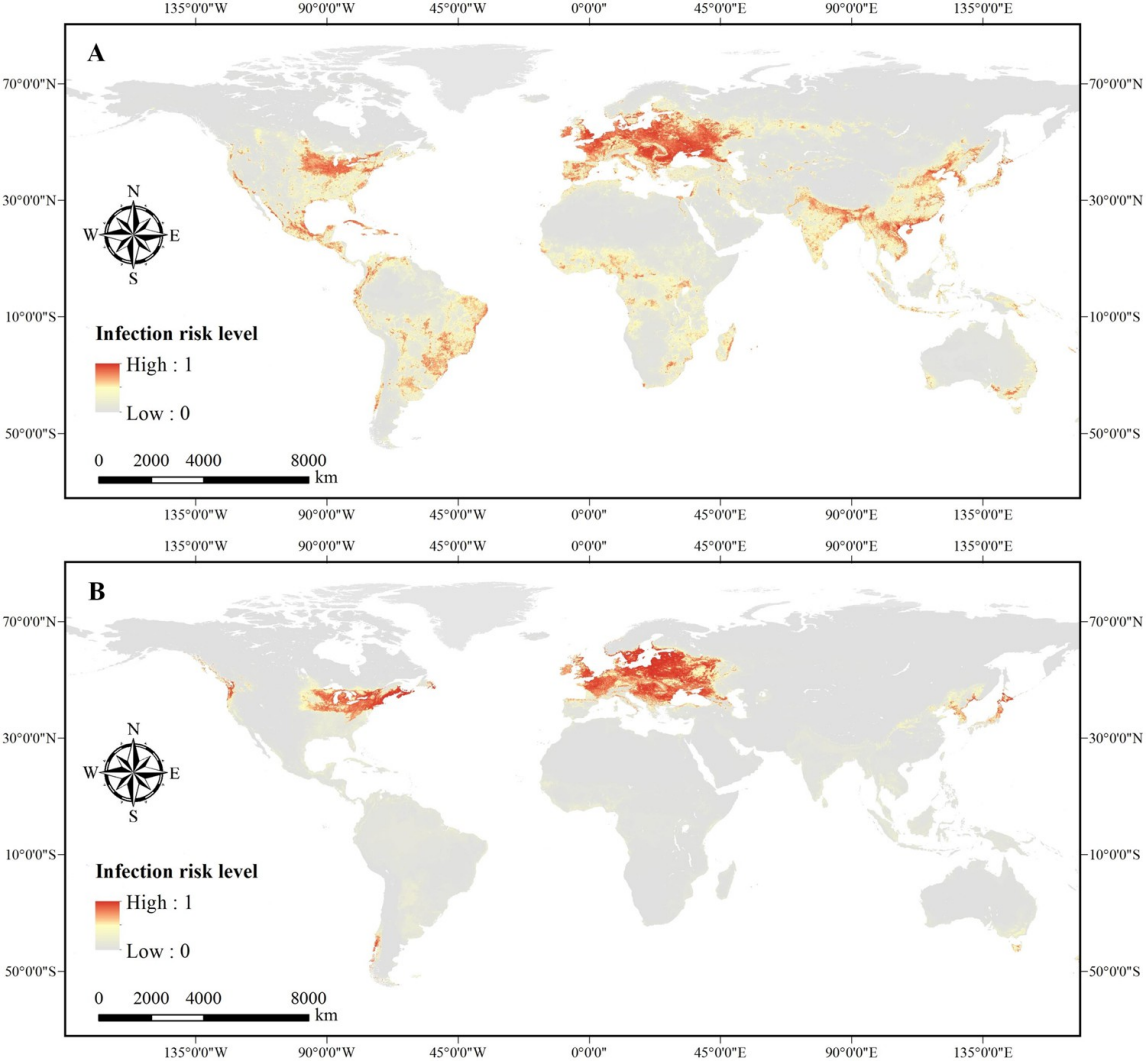
Maps of global spread risk level for African swine fever, ranging from 0 (grey) to 1 (red), which were derived from domestic pigs (A) and wild boars (B) respectively.

Maps showing the predicted global potential infection risk of ASF are presented in Fig 2. The potential high spread risky zones of ASF derived from domestic pigs are predicted to occur in all continents except Antarctica, with hot spots in western Europe, tropical and sub-tropical areas of Africa and South America, tropical and temperate North America, southern and eastern Asia, and coastal Oceania. By contrast, the distribution of predicted risk areas derived from wild boars will be confined to Europe, central North America (mainly concentrated in the United states), and relatively few areas in eastern Asia (parts of China, Korea and Japan). In total, the potential distribution of predicted risk areas derived from domestic pigs shows a wider geographical distribution than that derived from wild boars. Validation statistic index revealed that the ensemble 150 BRT models indicate a high performance in quantifying the spread risk level of ASF derived from domestic pigs (AUC-ROC of training dataset 10-fold-cross: 0.957 ± 0.003; AUC-ROC of validation dataset: 0.957 ± 0.007) and wild boars (AUC-ROC of training dataset 10-fold-cross: 0.992±0.001; AUC-ROC of validation dataset: 0.991±0.002). We also generated the predicted spread risk maps using the 95% confidence intervals of the ensemble BRT models, as shown in S1 and S2 Figs.

### The number of domestic pigs at risk

We calculated the number of domestic pigs located in an area at risk of ASF by combining the global predicted disease infection risk map with the fine-scale global domestic pig population surface. First, the threshold predicted risk values of 0.63 and 0.88 were adopted to convert our predicted infection risk maps (Fig 2) into binary surfaces (S3 Fig), which were determined to incorporate 90% of all the ASF case locations derived from domestic pigs and wild boars respectively. Every 5 km x 5 km pixel in the risk map with a value above the threshold value was considered at risk for ASF infection. Next, we multiplied the generated binary risk maps by the global domestic pig population surface, and finally we summed across all grids by continents (countries) to estimate the population at risk. Table 2 listed the domestic pig population (thousands) at risk of ASF transmission for each continent and the top five countries contributing to the potential domestic pig population at risk. We summed the swine populations at risk and have identified about 1,388 million (52% of the total swine population) swine globally living within areas that are suitable for ASF transmission. Asia contributes most of the pig population at risk globally, with 588.6 million head, of which China holds an important share (423 million). There are about 433 million pigs at risk in Europe, with half of those living in Germany, Spain, Poland, France and Demark. In the Americas, more than 340 million pigs live in the ASF risk zones, with approximately 48 percent of the population living in the United States. About 22 million pigs living in Africa are at risk of ASF, of which countries of Nigeria, Uganda and South Africa accounting for 50 percent. In Oceania, roughly 4.8 million pigs are living in risky areas for ASF, the largest proportion of which live in Australia (about 4 million).

**Table 2:** The domestic pig population (thousands) exist in the predicted potential risk areas for African swine fever within each continent and the top five countries contributing to these population at risk.

	Country	Population		Country	Population		Country	Population		Country	Population
**Scenario A.**											
**America(340,352)**	United States	163,471	**Asia (588,633)**	China	423,245	**Europe (433,094)**	Germany	59,676	**Oceania (4,796) & Africa (22,000)**	Nigeria	6,333
	Brazil	74,149		Vietnam	64,101		Spain	45,973		Australia	3,944
	Mexico	31,092		South Korea	19,488		Poland	40,260		Uganda	2,674
	Canada	24,195		India	17,893		France	38,533		South Africa	2,021
	Colombia	7,212		Japan	14,721		Denmark	33,611		Cameroon	1,739

## Discussion

ASF is sweeping the global pig industry [44]. However, the potential geographic extent of ASF infection zones is unknown, as are the risk factors associated with it. Based on a modelling framework, we paired the assembled contemporary records of ASF cases with a set of livestock, anthropogenic and habitat correlates to quantify the risk factors and the risk of potential spread of ASF worldwide. Given the high cost of active surveillance and the limited resources of veterinary services in some countries [45, 46], risk-based surveillance can save costs and help allocate limited resources reasonably to maximize utility. This study provided an important baseline for monitoring the risk of spread of ASF by estimating a possible range of infection zones and the number of domestic pigs in at-risk areas.

The predicted infection risk maps reveal that ASF has the potential to be spread to many parts of the world, especially in the northern hemisphere. For instance, in the United States, the potential geographic extent of ASF derived from domestic pigs or wild boars are mainly distributed in several states in the northeast, such as Wisconsin, Illinois, Indiana, Michigan, Ohio, Pennsylvania and New Hersey. The potential geographic extent of ASF derived from domestic pigs is different from that for wild boars. The former is influenced strongly by density of domestic pigs, while the latter is associated with natural habitat covariates. In addition, the pixel-level uncertainty of the ensemble BRT models was also quantified using the standard deviation method, as shown in S4 and S5 Figs. The uncertainty maps suggest that the uncertainty of the ensemble BRT approach is low as a whole.

Several previous literatures have linked climate-related covariates to the presence of ASF [23]. These climate factors affect the habitat distribution of wild boars and soft ticks [24, 33–36], which could reflect the spatial distribution of the diseases to a certain extent. Compared with these studies, we used more abundant spatial covariates (i.e., anthropogenic and natural habitat factors), and combined with BRT modeling procedure to specifically mine the complex relationships between spatial covariates and the presence of African swine fever, as presented in S6 and S7 Figs. For example, domestic pig population is positively associated with the occurrence of ASF in domestic pigs, while there is a negative relationship between domestic pig population and ASF derived from wild boars.

By combining the domestic pig population maps with the estimated risk maps, we estimated that nearly half of the world’s domestic pigs (1.388 billion) is raised in the predicted high-risk zones. Given there are no commercial vaccines available to eradicate ASF, improving biosafety in pig farms is currently the best way to prevent the disease [47]. For the endemic zones, it is suggested that all pigs in the case farms should be slaughtered quickly, and safe disposal should be applied to all dead and slaughtered pigs and relevant products. The contaminated materials (i.e., excreta, leftover feed and sewage) should be cleaned and disposed of safely. The importation and exportation of susceptible animals and related products should be suspended when needed. For the areas belonging to the predicted high-risk zones, it is necessary to strengthen biosafety education to not only pig farmers but the public. This is important for the prevention of ASF and to improve the awareness of biosafety in pig farms. For example, protection measures should be taken to prohibit feeding of unheated swill to pigs, and avoid domestic pigs having contact with wild boars and soft ticks. In addition, quarantine should be carried out in pig farms during introduction, to reduce the risk of ASF.

There are some limitations in this study. First, considering not all continents have the same sensitive surveillance system, the notified cases in several zones (i.e., Africa) may be under-estimated, which may bring some uncertainty to the analysis. Secondly, the estimated spread risk maps can be interpreted to predict the potential geographic extent of ASF in the world, rather than where the disease will be spread in the future. A strong spatio-temporal prediction of the geographic distribution of ASF spread requires data on the movement of live pigs and pork products, farm management and migration of wild boars, which were not yet available when conducting this study. For future work, we will cooperate with multiple business units to collect more data (i.e., the movement of pigs and pork products, and farm management) to improve the model. Besides, we will combine the profits and costs of domestic pig production to evaluate the economic losses that ASF may lead to on country and global scale.

## Supporting information

S1 Fig The global spread risk level for African swine fever derived from domestic swine. (TIF)

S2 Fig The global spread risk level for African swine fever derived from wild boar. (TIF)

S3 Fig Binary maps of global spread risk level for African swine fever. (TIF)

S4 Fig The uncertainty of the predicted risk level derived from domestic swine based on the ensemble BRT model. (TIF)

S5 Fig The uncertainty of the predicted risk level derived from wild swine based on the ensemble BRT model. (TIF)

S6 Fig The relationships between spatial covariates and the presence of African swine fever derived from domestic swine over all boosted regression tree (BRT) ensembles. (TIF)

S7 Fig The relationships between spatial covariates and the presence of African swine fever derived from wild boar over all boosted regression tree (BRT) ensembles. (TIF)

S1 Table Spatial predictor variables adopted in this study. (DOCX)

S2 Table Correlation matrix between covariate variables used in BRT ensembles trained on domestic swine samples. (DOCX)

S3 Table Correlation matrix between covariate variables used in BRT ensembles trained on wild boar samples. (DOCX)
