# Supplementary figures and images for: Quantifying risk factors and potential geographic extent of African swine fever across the world
Source: PLoS One. 2022 Apr 21;17(4):e0267128. doi: 10.1371/journal.pone.0267128 (PMC9022809; doi:10.1371/journal.pone.0267128)

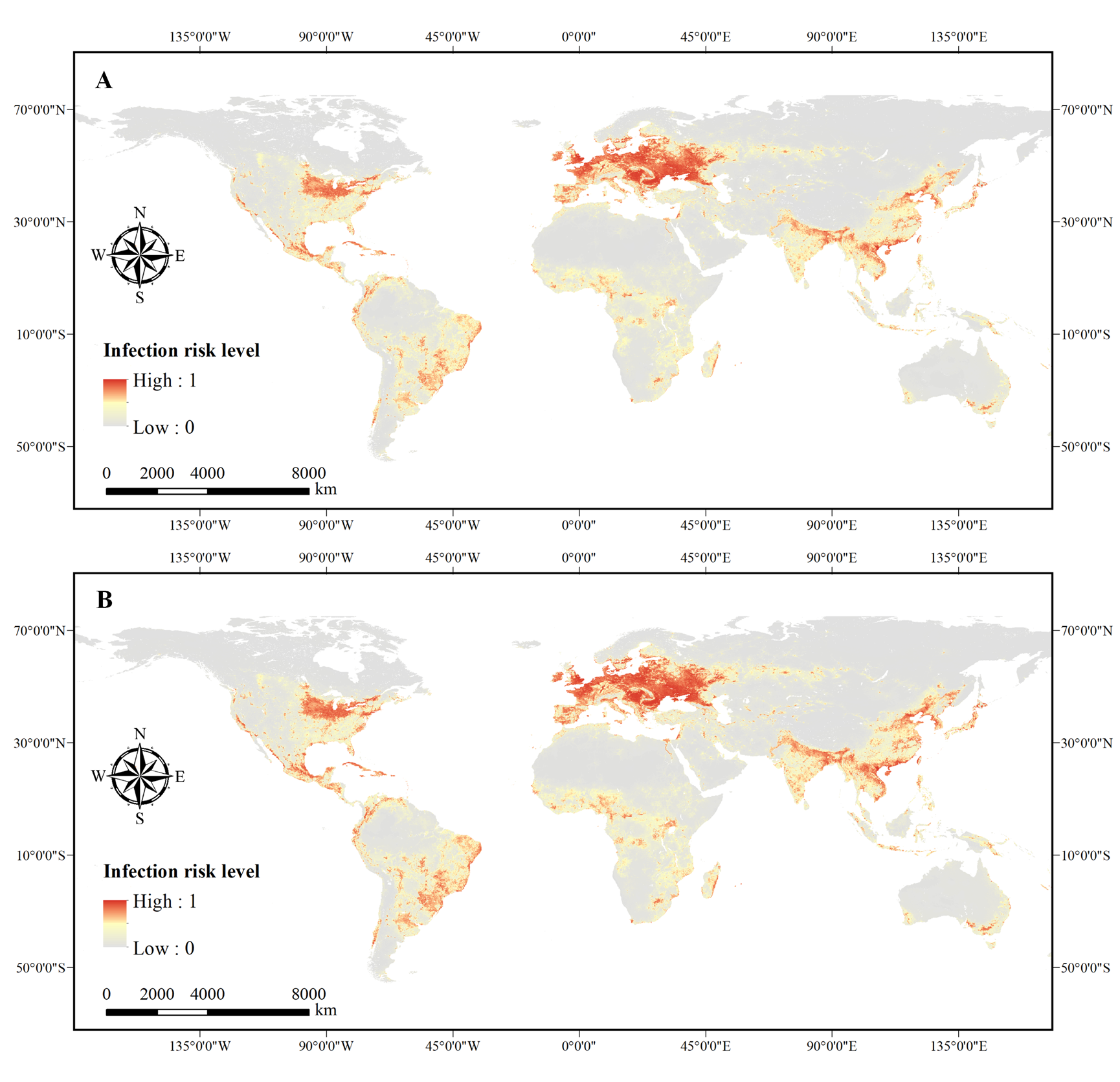

Supplement: S1 Fig — (TIF) [file pone.0267128.s001.tif]

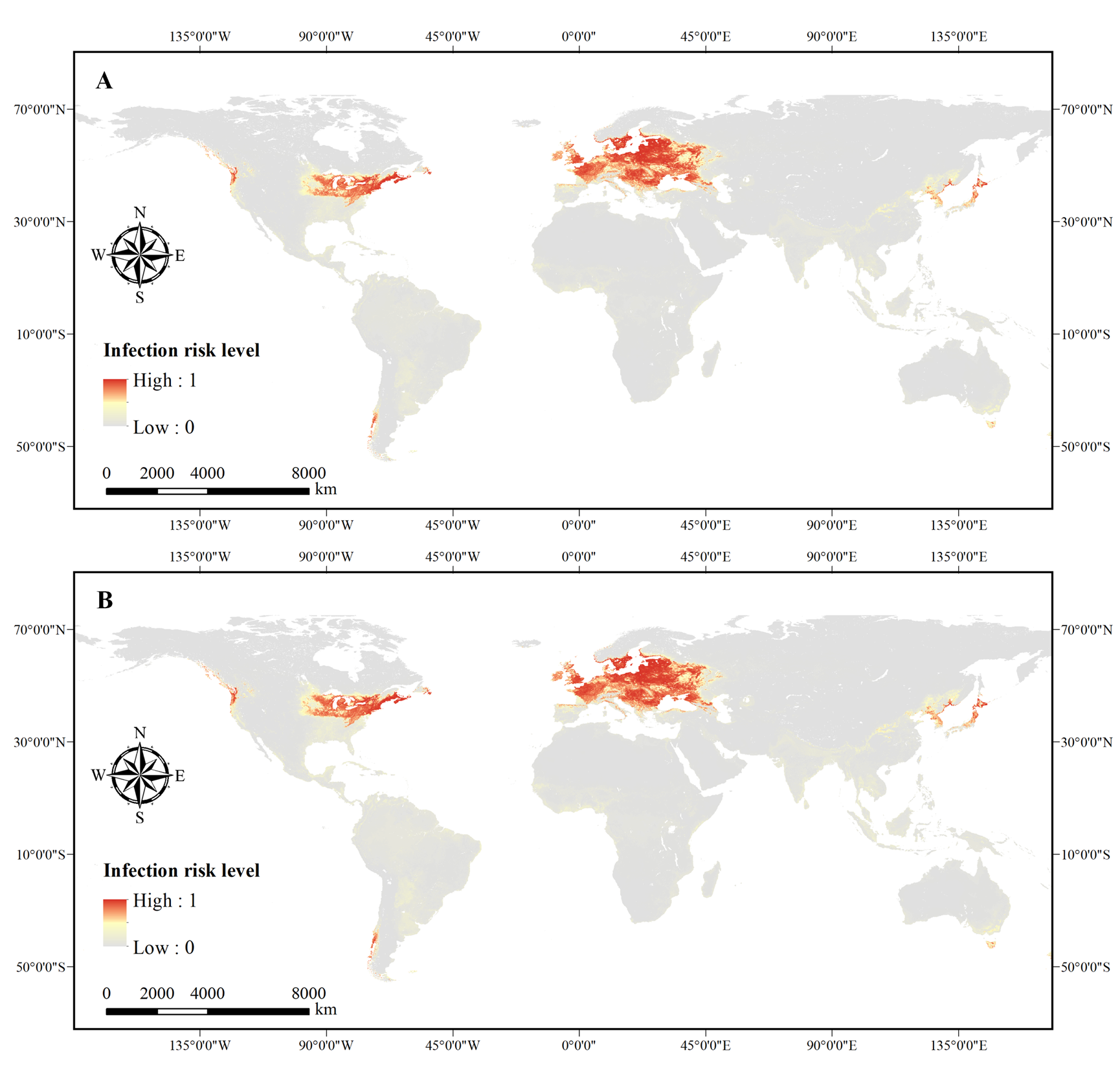

Supplement: S2 Fig — (TIF) [file pone.0267128.s002.tif]

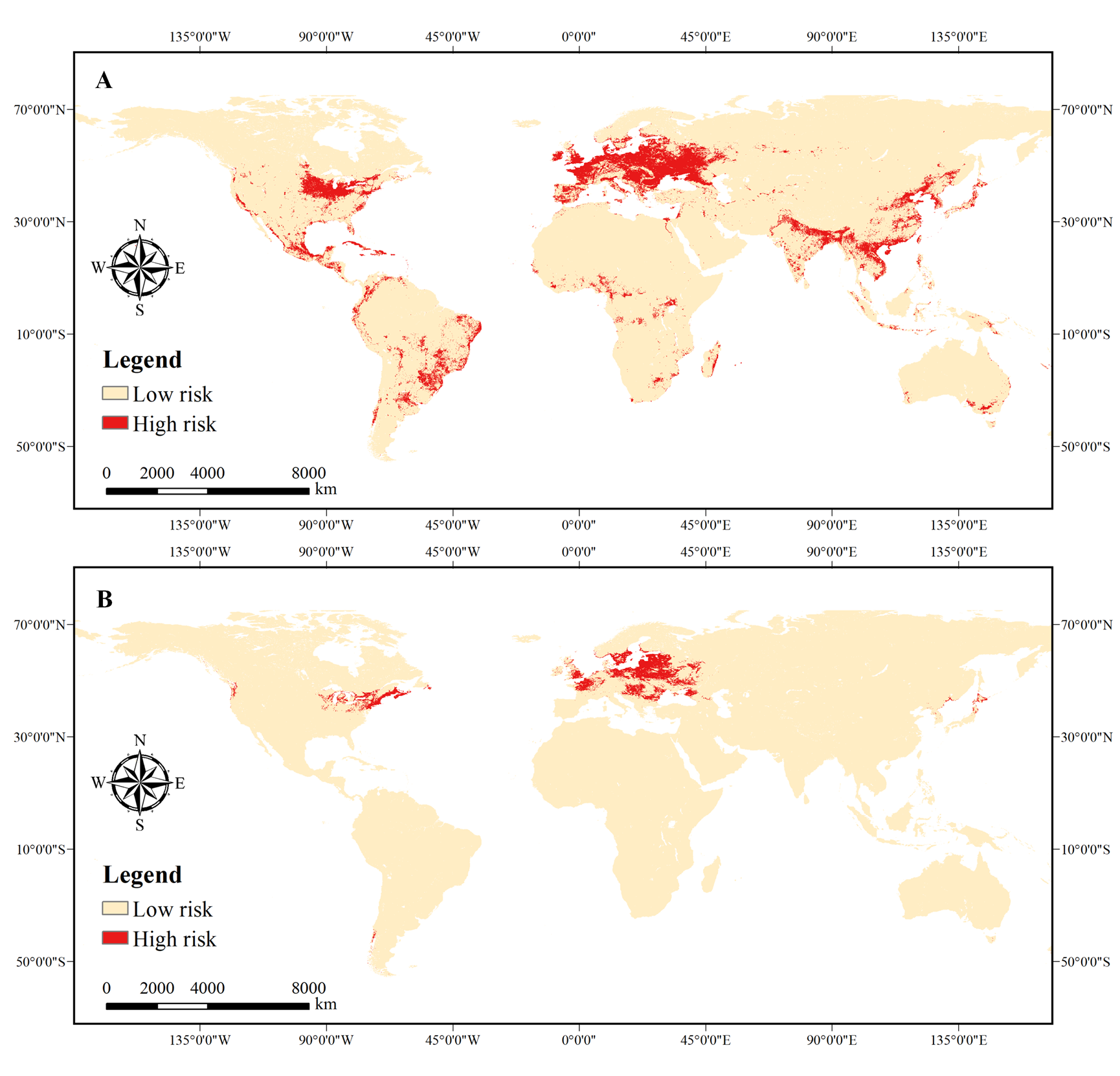

Supplement: S3 Fig — (TIF) [file pone.0267128.s003.tif]

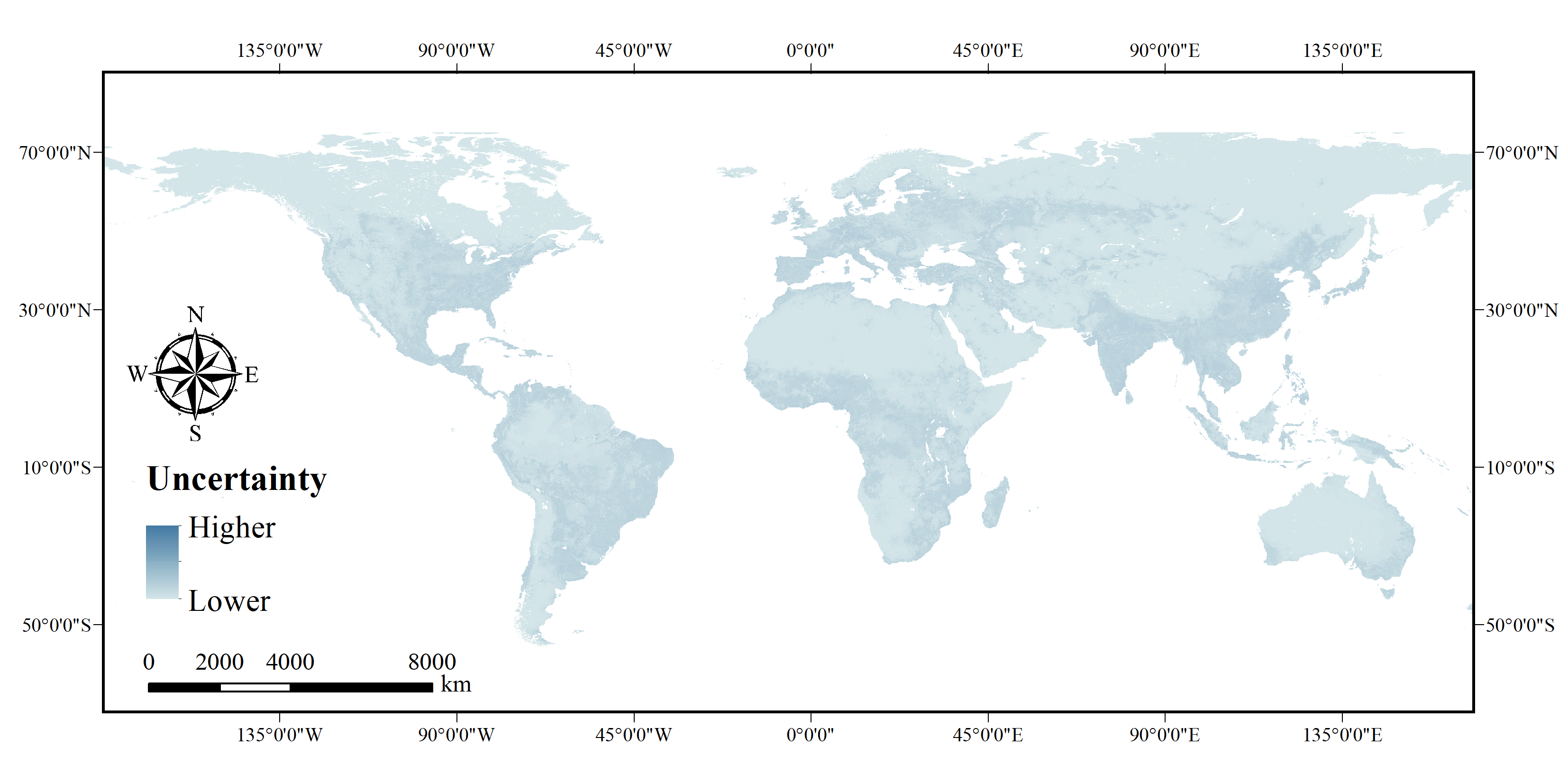

Supplement: S4 Fig — (TIF) [file pone.0267128.s004.tif]

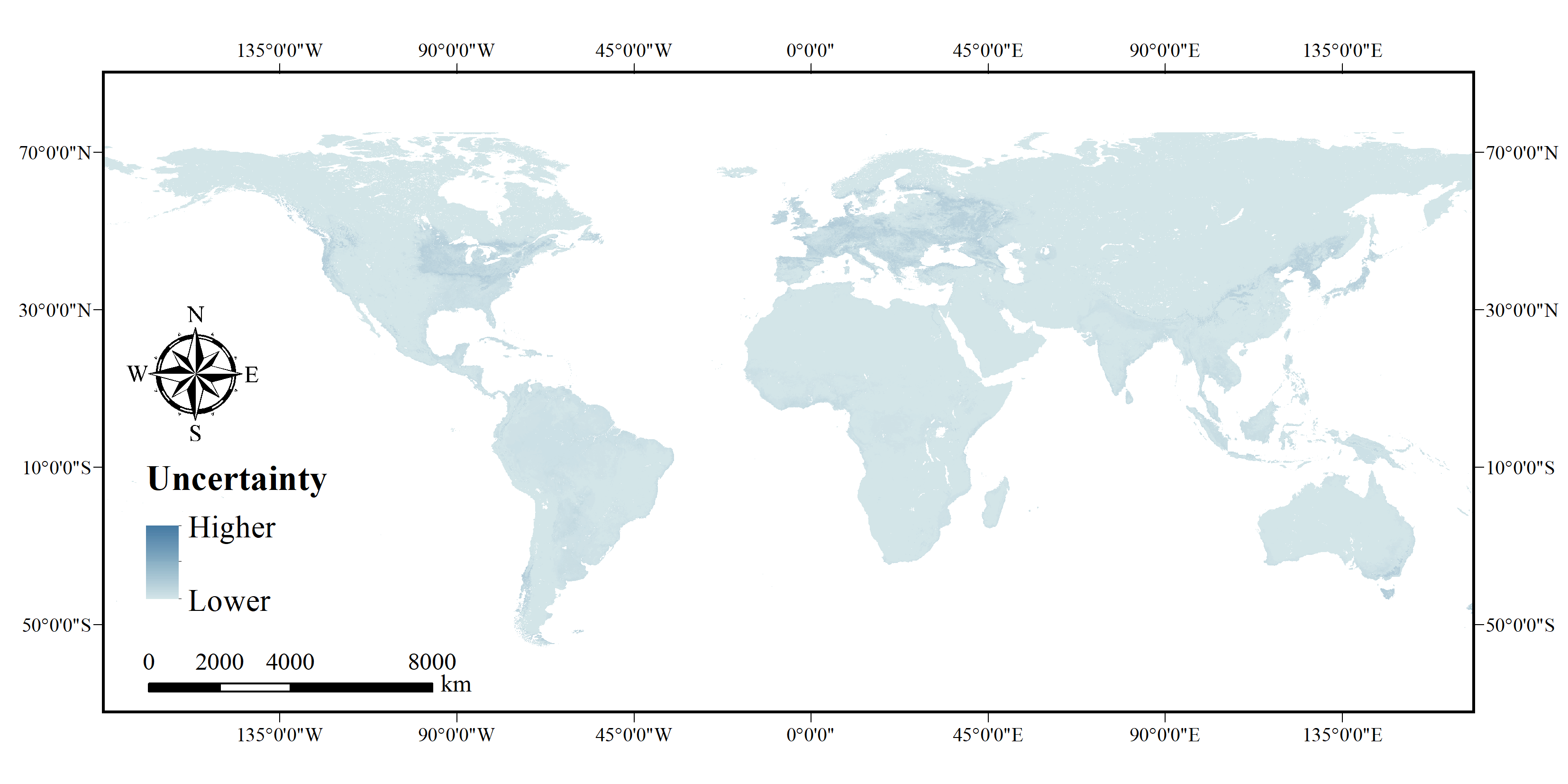

Supplement: S5 Fig — (TIF) [file pone.0267128.s005.tif]

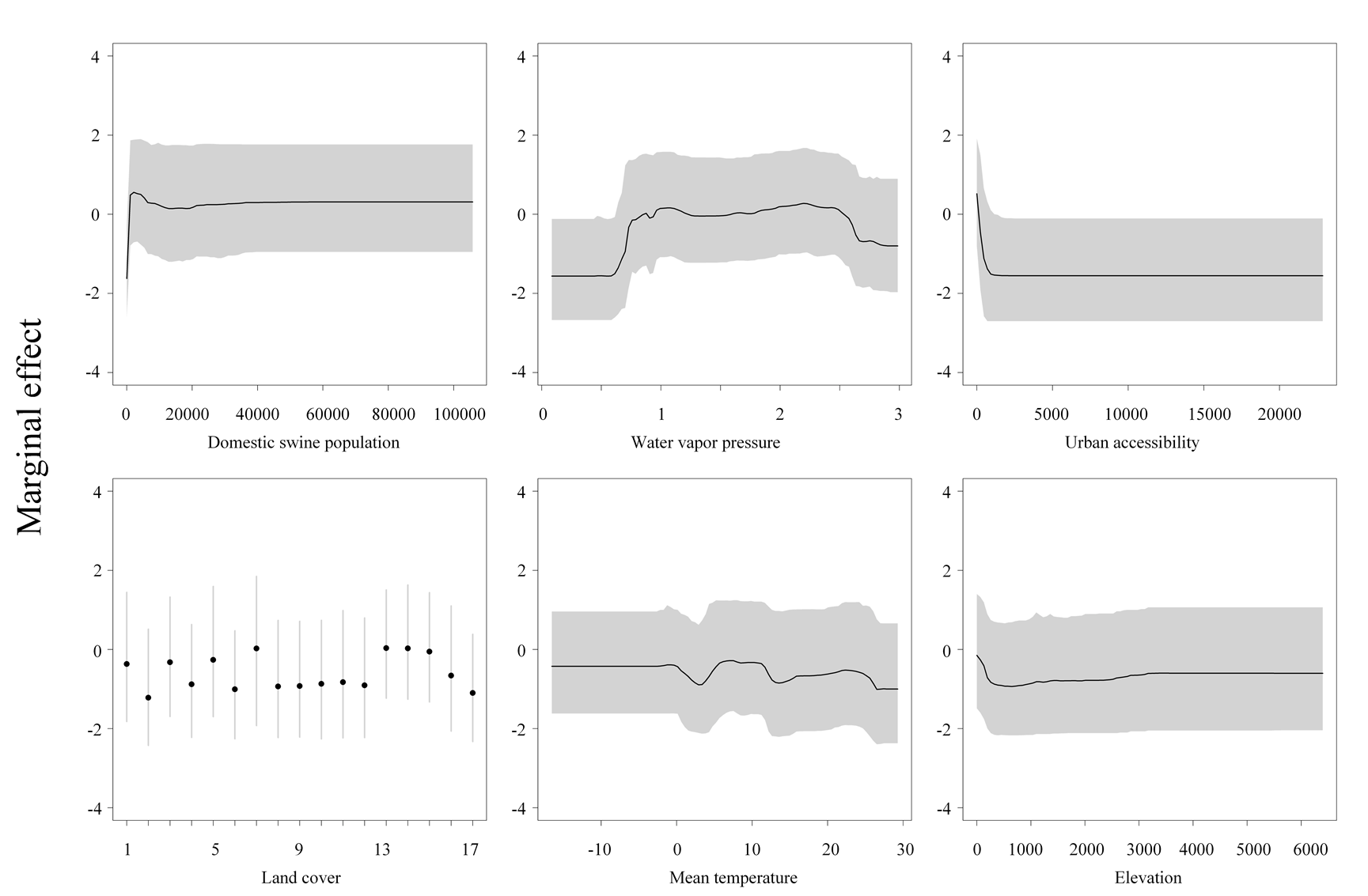

Supplement: S6 Fig — (TIF) [file pone.0267128.s006.tif]

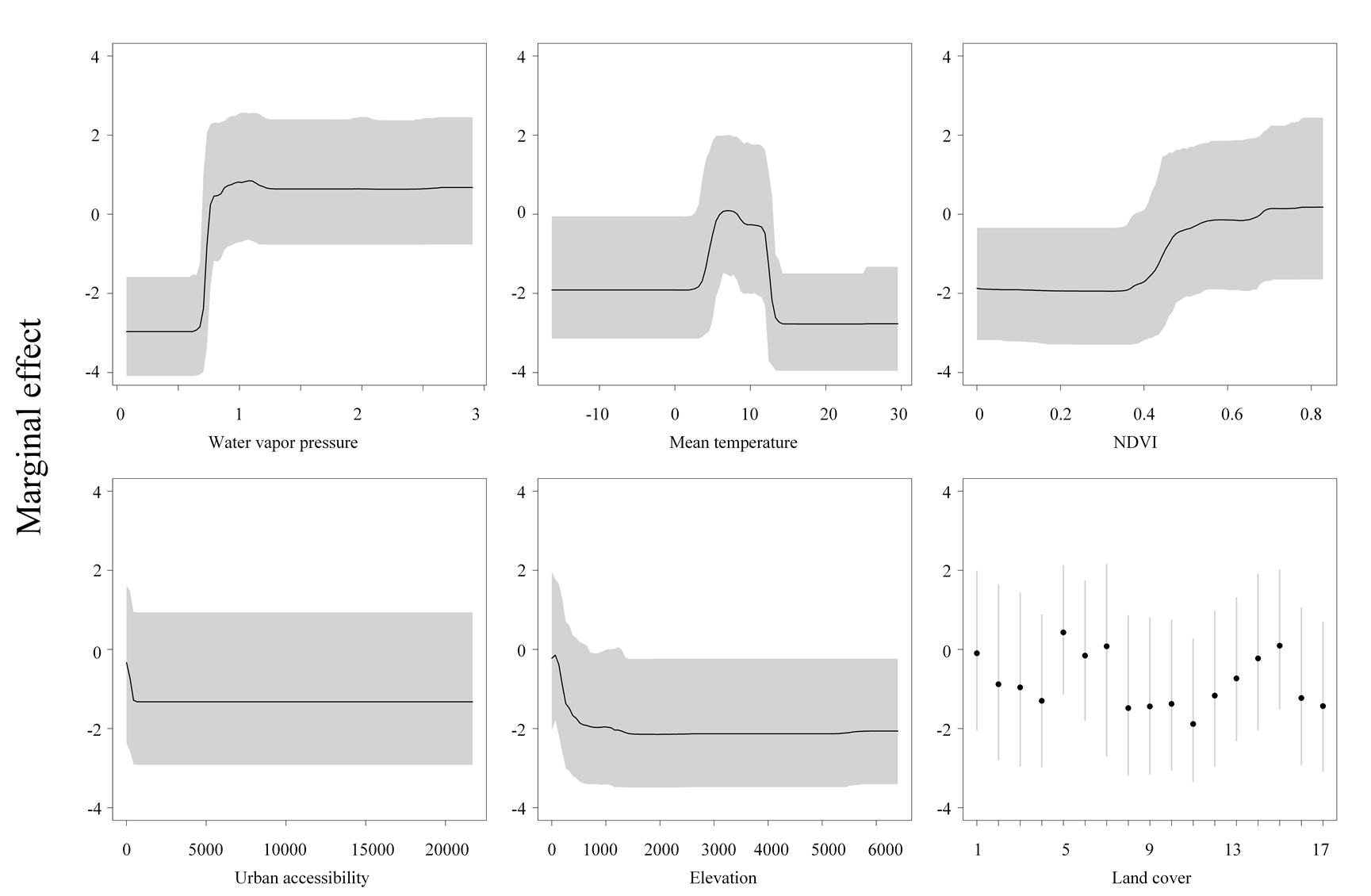

Supplement: S7 Fig — (TIF) [file pone.0267128.s007.tif]
