# Supplementary material for: Quantifying risk factors and potential geographic extent of African swine fever across the world
Source: PLoS One. 2022 Apr 21;17(4):e0267128. doi: 10.1371/journal.pone.0267128 (PMC9022809; doi:10.1371/journal.pone.0267128)
Supplement: S1 Table — (DOCX) [file pone.0267128.s008.docx]

**S1 Table. Spatial predictor variables adopted in this study.**

| **Factors** | **Parameters** | **Data sources** |
| --- | --- | --- |
| Livestock | Domestic swine population | Food and Agriculture Organization |
| Anthropogenic | Urban accessibility | European Commission Joint Research Centre Global Environment Monitoring Unit |
|  | Population density | Socioeconomic Data and Applications Center, NASA |
|  | Nighttime lights | The Earth Observation Group, NOAA |
| Habitat | Land cover | NASA's Earth Observatory Group |
|  | Elevation | Consultative Group on International Agricultural Research (CGIAR) Consortium for Spatial Information |
|  | Mean temperature | WorldClim database, version 2 |
|  | Water vapor pressure |  |
|  | Annual cumulative precipitation |  |
|  | Normalized difference vegetation index | Global Inventory Modeling and  Mapping Studies Group |
