# Supplementary material for: Quantifying risk factors and potential geographic extent of African swine fever across the world
Source: PLoS One. 2022 Apr 21;17(4):e0267128. doi: 10.1371/journal.pone.0267128 (PMC9022809; doi:10.1371/journal.pone.0267128)
Supplement: S2 Table — (DOCX) [file pone.0267128.s009.docx]

**S2 Table. C****orrelation matrix** **between covariate variables used in BRT ensembles trained on domestic swine samples.**

|  | NDVI | LC | NTL | PD | E | UA | DSP | MT | ACP | WVP |
| --- | --- | --- | --- | --- | --- | --- | --- | --- | --- | --- |
| NDVI | 1 | -0.17 | 0.093 | 0.053 | -0.266 | -0.451 | 0.123 | 0.611 | 0.661 | 0.656 |
| LC | -0.17 | 1 | 0.203 | 0.105 | -0.034 | -0.174 | 0.124 | 0.177 | -0.247 | -0.019 |
| NTL | 0.093 | 0.203 | 1 | 0.532 | -0.132 | -0.194 | 0.202 | 0.173 | 0.067 | 0.136 |
| PD | 0.053 | 0.105 | 0.532 | 1 | -0.063 | -0.098 | 0.34 | 0.186 | 0.181 | 0.23 |
| E | -0.266 | -0.034 | -0.132 | -0.063 | 1 | 0.396 | -0.105 | -0.127 | -0.087 | -0.181 |
| UA | -0.451 | -0.174 | -0.194 | -0.098 | 0.396 | 1 | -0.134 | -0.466 | -0.155 | -0.32 |
| DSP | 0.123 | 0.124 | 0.2s02 | 0.34 | -0.105 | -0.134 | 1 | 0.321 | 0.321 | 0.425 |
| MT | 0.611 | 0.177 | 0.173 | 0.186 | -0.127 | -0.466 | 0.321 | 1 | 0.629 | 0.778 |
| ACP | 0.661 | -0.247 | 0.067 | 0.181 | -0.087 | -0.155 | 0.321 | 0.629 | 1 | 0.743 |
| WVP | 0.656 | -0.019 | 0.136 | 0.23 | -0.181 | -0.32 | 0.425 | 0.778 | 0.743 | 1 |

Note: NDVI (Normalized difference vegetation index): VIF = 2.613; LC (Land cover): VIF = 1.444; NTL (Nighttime lights): VIF = 1.489; PD (Population density): VIF = 1.557; E (Elevation): VIF = 1.347; UA (Urban accessibility): VIF = 1.823; DSP (Domestic swine population): VIF = 1.433; MT (Mean temperature): VIF = 7.659; ACP (Annual cumulative precipitation): VIF = 5.466; WVP (Water vapor pressure): VIF = 9.594.
