# Supplementary material for: Quantifying risk factors and potential geographic extent of African swine fever across the world
Source: PLoS One. 2022 Apr 21;17(4):e0267128. doi: 10.1371/journal.pone.0267128 (PMC9022809; doi:10.1371/journal.pone.0267128)
Supplement: S3 Table — (DOCX) [file pone.0267128.s010.docx]

**S3 Table. Correlation matrix between covariate variables used in BRT ensembles trained on wild boar samples.**

|  | NDVI | LC | NTL | PD | E | UA | DSP | MT | ACP | WVP |
| --- | --- | --- | --- | --- | --- | --- | --- | --- | --- | --- |
| NDVI | 1 | -0.118 | 0.142 | 0.064 | -0.375 | -0.546 | 0.14 | 0.564 | 0.675 | 0.615 |
| LC | -0.118 | 1 | 0.171 | 0.102 | 0.087 | -0.077 | 0.134 | 0.22 | -0.196 | 0.023 |
| NTL | 0.142 | 0.171 | 1 | 0.559 | -0.11 | -0.168 | 0.132 | 0.155 | 0.026 | 0.111 |
| PD | 0.064 | 0.102 | 0.559 | 1 | -0.047 | -0.084 | 0.229 | 0.126 | 0.077 | 0.129 |
| E | -0.375 | 0.087 | -0.11 | -0.047 | 1 | 0.416 | -0.057 | -0.137 | -0.144 | -0.196 |
| UA | -0.546 | -0.077 | -0.168 | -0.084 | 0.416 | 1 | -0.129 | -0.527 | -0.309 | -0.406 |
| DSP | 0.14 | 0.134 | 0.132 | 0.229 | -0.057 | -0.129 | 1 | 0.26 | 0.265 | 0.308 |
| MT | 0.564 | 0.22 | 0.155 | 0.126 | -0.137 | -0.527 | 0.26 | 1 | 0.556 | 0.759 |
| ACP | 0.675 | -0.196 | 0.026 | 0.077 | -0.144 | -0.309 | 0.265 | 0.556 | 1 | 0.697 |
| WVP | 0.615 | 0.023 | 0.111 | 0.129 | -0.196 | -0.406 | 0.308 | 0.759 | 0.697 | 1 |

Note: NDVI (Normalized difference vegetation index): VIF = 2.751; LC (Land cover): VIF = 1.324; NTL (Nighttime lights): VIF = 1.548; PD (Population density): VIF = 1.521; E (Elevation): VIF = 1.430; UA (Urban accessibility): VIF = 1.921; DSP (Domestic swine population): VIF = 1.188; MT (Mean temperature): VIF = 6.793; ACP (Annual cumulative precipitation): VIF = 4.751; WVP (Water vapor pressure): VIF = 9.057.
